# Frequency dependency of therapeutic efficacy in dorsal root ganglion stimulation for neuropathic pain

**DOI:** 10.1007/s00701-022-05161-6

**Published:** 2022-02-26

**Authors:** G. S. Piedade, S. Gillner, P. S. McPhillips, J. Vesper, P. J. Slotty

**Affiliations:** 1 Department of Neurosurgery, Heinrich-Heine-Universität Düsseldorf, Moorenstr. 5, 40225 Düsseldorf, Germany; 2 Department of Functional Neurosurgery and Stereotaxy, Heinrich-Heine-Universität Düsseldorf, Moorenstr. 5, 40225 Düsseldorf, Germany

**Keywords:** Neuropathic pain, Dorsal root ganglion stimulation, Frequency

## Abstract

**Background:**

The influence of the stimulation frequency on the outcomes of dorsal root ganglion stimulation (DRG-S) to treat pain is not well understood. It is assumed that specific neural components dedicated to different tasks in the DRG can be preferably influenced at specific frequencies. The identification of frequencies designed for the type of pain and the ratio of neuropathic versus nociceptive pain might improve overall pain control and open new indications in DRG-S.

**Method:**

We report on a randomized double-blind clinical trial with a crossover design. Patients with a permanent DRG-S system underwent phases of stimulation with 20 Hz, 40 Hz, 60 Hz, 80 Hz, and sham in a randomized order. Each phase lasted for 4 days and was followed by a 2-day washout period. Pain intensity and quality of life were assessed with visual analog scale (VAS), McGill Pain Questionnaire (MPQ), EQ-5D, and Beck Depression Inventory (BDI). Analgesics intake was assessed.

**Results:**

Overall 19 patients were included in the study. CRPS was the most frequent pain etiology (7). Five patients had a PainDetect score of 12 or lower at baseline. The mean VAS before the system was implanted was 8.6 and 3.9 at the baseline. Pain intensity was reduced to 3.7 by the stimulation with 20 Hz but increased with higher frequencies reaching 5.8 at 80 Hz. A significant difference among the groups was shown over all variables examined (VAS, MPQ, EQ-5D, BDI). The best results were seen at 20 Hz for all variables, including the smallest increase in pain medication consumption.

**Conclusions:**

The choice of the stimulation frequency shows a clear influence on pain reduction and quality of life. Lower stimulation frequencies seem to be most effective in neuropathic pain. Further studies are required to determine whether specific frequencies should be preferred based on the condition treated.

## Introduction

Dorsal root ganglion (DRG) stimulation has been effectively used in the treatment of neuropathic pain of different etiologies. In neuropathic pain, spontaneous firing as a consequence of lower action potential thresholds can be observed in the DRG neurons [12]. Different stimulation frequencies could lower this abnormal activity with different intensities by readjusting the action potential threshold. In a traditional view of “stimulation dose,” patients requiring more pain relief would respond to a higher total electrical energy delivery, which is dependent on current, pulse width, and stimulation frequency [11]. However, recent studies have shown that DRG-S with lower frequencies—and therefore with lower total energy delivery—could be an effective alternative. A sub-analysis of the ACCURATE study [3] reported paresthesia-free subjects using DRG-S that achieved similar pain relief with lower amplitudes and frequencies [10]. Koetsier et al. were able to show a delayed washout effect of DRG-S in the treatment of painful diabetic neuropathy in rats [8]. Chapman et al. reported a case series with tapering of stimulation frequencies in twenty patients with refractory back pain down to 4 Hz and reported sustained pain relief [2].

It is assumed that specific neural components of the DRG can be influenced in a targeted manner by the selection of different frequencies and that different pain patterns can be optimally treated with different frequencies. Little is known about the effect of stimulation frequency over the clinical outcomes of DRG-S. We report on the first randomized double-blind clinical trial testing mid-frequency DRG-S in patients with neuropathic pain.

## Materials and methods

Patients aged above 18 years old with a DRG stimulation system implanted and followed-up at the Department of Neurosurgery of the Heinrich-Heine-University Düsseldorf were invited to participate in the study. Written informed consent was obtained. Individuals were excluded from the trial in case of further significant pain that might confound the study assessments. Nineteen patients participated in the study. The study was approved by the Ethics Committee of the Medical Faculty under the number 2020–1120 and was registered at the German Clinical Trials Register (DRKS) under DRKS00022557.

Patients were evaluated for neuropathic pain with PainDetect (0–38 points) at the baseline. All patients tested five different stimulation parameter settings in a randomized order: stimulation frequencies of 20 Hz, 40 Hz, 60 Hz, 80 Hz, and sham stimulation. Sham means amplitude set at 0.025 mA, the minimum amplitude allowed, so that the IPG indicates to the patient stimulation on, but delivers only ineffective stimulation. Patients were programmed at subthreshold for each tested frequency; amplitude was corrected in each case. Patients and investigators were blinded, and a study nurse had access to unblinded data. Each stimulation parameter setting was tested for 4 days and was followed by a 2-day washout period. The stimulation parameters were programmed in advance by a study nurse and were randomly changed by the patients each week at home. The stimulation amplitude was programmed to subthreshold levels individually for each frequency. At the end of each phase, the patients were interviewed by phone and completed numbered questionnaires.

At baseline, VAS and clinical parameters were assessed, and pre DRG-S pain data was collected from charts. During the study, patients underwent assessment of pain intensity and quality using the visual analog scale and McGill Pain Questionnaire (MPQ, 0–78 points), of quality of life using EQ-5D (Index 0–1), and of the prevalence of depression using the Beck Depression Inventory (BDI, 0–63 points). Any additional intake of analgesics was documented by the patients.

### Statistical analysis

Patients’ demographics were analyzed using descriptive statistics and presented as frequency and percentage for categorical variables, and as numbers, means, minima, maxima, and standard deviations (SD) for continuous variables. Statistical analysis was performed using SPSS 19 software (IBM Cooperation, USA) and GraphPad Prism 8.0.2.

Repeated measurement one-way ANOVA was used for comparison between baseline data and measurements at the different frequency settings applying Tukey’s multiple comparison test. An alpha error of 0.05 was considered significant, and 0.01 was considered highly significant.

## Results

A total number of 19 patients participated in the study. The mean age was 53 years (range: 25–80) and the patients were using DRG-S for a mean of 17.2 months (range: 4–102). The most common pain etiology was chronic regional pain syndrome (CRPS) (7 subjects), followed by postsurgical pain after implantation of joint prosthesis (4), postherpetic neuralgia (3), nerve injury after resection of neurinomas (2), traumatic nerve injury (2), and diabetic polyneuropathy (1). Fourteen patients had a PainDetect Score of 12 or higher (76.7%), indicating higher probability of neuropathic pain. Patients reported a mean VAS of 8.6 (SD 1.0) before the implantation of the DRG-S system and a mean baseline VAS of 3.9 (SD 1.9). All patients had already been programmed in the clinical routine and had reached a stable therapeutic response. All patients had a stimulation frequency of 20 Hz at study start.

Even at subthreshold level with corrected amplitude, some patients experienced at higher frequencies a change in the paresthesia field. Amplitude was reduced in these cases. No patient had painful paresthesia nor motor stimulation.

**Fig. 1:**
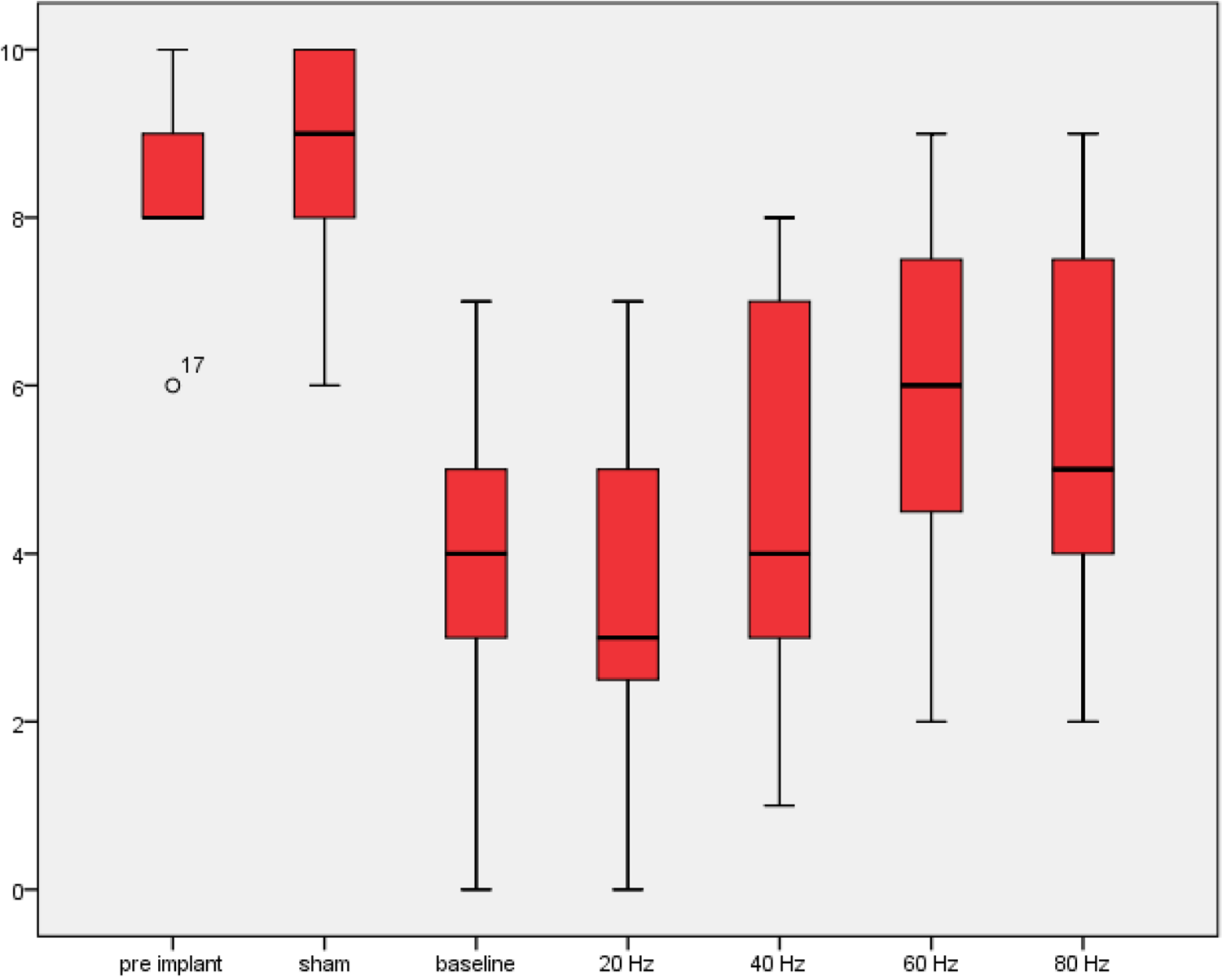
Mean VAS pre DRG-S, under sham stimulation, at baseline and under 20, 40, 60, and 80 Hz

Results for mean VAS for 20 Hz, 40 Hz, 60 Hz, 80 Hz, and sham stimulation were 3.7 (SD 1.9), 4.9 (SD 2.2), 5.8 (SD 1.9), 5.8 (SD 1.9), and 8.6 (SD 1.3) respectively (Table 1). 20 Hz achieved significantly lower pain intensity than 40 Hz (*p* = 0.004) and any other tested stimulation parameters (*p* < 0.001). 40 Hz did not result in significantly better results than 60 Hz (*p* = 0.086), nor did 60 Hz have lower pain intensities than 80 Hz (*p* = 0.695) (Fig. 1). Although the overall trend and statistics favor lower stimulation frequencies, two patients preferred higher stimulation frequencies and reported better pain control. In both cases, amplitude remained at the necessary level for subthreshold stimulation.

**Table 1:** Pain intensity under stimulation frequencies of 20 Hz, 40 Hz, 60 Hz, 80 Hz, and sham stimulation. *CRPS*, complex regional pain syndrome; *SD*, standard deviation

No	Age	Pain etiology	PainDetect	VAS pre DRG-S	VAS baseline	VAS 20 Hz	VAS 40 Hz	VAS 60 Hz	VAS 80 Hz	VAS Sham
1	26	Postherpetic neuralgia	17	8	4	4	8	6	4	8
2	59	CRPS	9	9	7	5	7	7	7	8
3	59	Postsurgical after implantation of joint prothesis	19	10	5	4	4	5	6	10
4	38	Nerve injury after neuroma resection	12	8	3	2	3	2	2	8
5	60	Postsurgical after implantation of joint prothesis	19	8	2	2	3	4	4	7
6	53	Postherpetic neuralgia	5	8	0	0	2	4	4	6
7	35	CRPS	24	9	3	3	1	9	7	9
8	70	Postherpetic neuralgia	19	8	1	2	5	8	8	8
9	65	Postsurgical after implantation of joint prothesis	14	8	5	5	7	8	8	9
10	80	CRPS	9	8	3	3	3	4	5	7
11	71	Traumatic nerve injury	9	8	3	3	4	5	5	7
12	40	CRPS	32	10	6	7	8	8	8	10
13	56	CRPS	13	8	4	3	7	7	9	9
14	35	CRPS	34	10	5	4	7	7	5	10
15	74	Diabetic polyneuropathy	15	6	4	3	4	5	4	10
16	48	Postsurgical after herniotomy	12	9	7	7	7	5	5	8
17	25	CRPS	20	9	7	7	7	6	8	9
18	50	Nerve injury after neuroma resection	16	10	4	5	4	8	7	10
19	55	Postsurgical after implantation of joint prothesis	7	9	2	2	3	4	4	10
		Mean (SD)	16 (7.9)	8.57 (1.01)	3.94 (1.98)	3.73 (1.91)	4.94 (2.19)	5.89 (1.88)	5.78 (1.93)	8.57 (1.26)

The same trend was seen with the McGill Pain Questionnaire, which resulted in 30.8 (SD 15.8), 33.1 (SD 17.3), 35.9 (SD 16.9), 36.3 (SD 14.2), and 46.5 (SD 17.2) points. In this case, statistical significance was only achieved when comparing MPQ results of 20 Hz and 80 Hz (*p* = 0.047). When analyzing quality of life, EQ-5D indexes were 0.76 (SD 0.16), 0.69 (SD 0.26), 0.59 (SD 0.30), 0.58 (SD 0.30), and 0.24 (SD 0.37). The index for 20 Hz was not significantly higher than for 40 Hz (*p* = 0.071), but than for 60 Hz and 80 Hz (*p* = 0.001).

Beck Depression Inventory resulted for the same groups 9.9 (SD 7.8), 10.8 (SD 7.1), 11.9 (SD 8.9), 13.6 (SD 8.7), and 15.5 (SD 10.2) points. Under 20 Hz, BDI was not significantly lower than under 40 Hz (*p* = 0.19), but under 60 Hz (*p* = 0.033) and 80 Hz (*p* = 0.005). Table 2 shows comprehensive data with the mean difference and statistical significance.

**Table 2:** Mean difference between baseline data and treatment groups adjusted with Tukey’s multiple comparison. No EQ5D, BDI, and MGPQ data is available at baseline and only VAS data is available pre DRG-S implantation. *Significant (< 0.05); **Highly significant (< 0.01). n.s., not significant

		pre DRG-S	Baseline	20 Hz	40 Hz	60 Hz	80 Hz	Sham
pre DRG-S	VAS		4.632 (**)	4.842 (**)	3.632 (**)	2.684 (**)	2.789 (**)	0.000 (n.s.)
	MGPQ							
	EQ5D							
	BDI							
Baseline	VAS	4.632 (**)		0.210 (n.s.)	− 1.000 (n.s.)	− 1.947 (*)	− 1.842 (*)	− 4.632 (**)
	MGPQ							
	EQ5D							
	BDI							
20 Hz	VAS	4.842 (**)	0,210 (n.s.)		− 1.211 (*)	− 2.158 (**)	− 2.053 (**)	− 4.842 (**)
	MGPQ				− 2.263 (n.s.)	− 5.053 (n.s.)	− 5.474 (*)	− 15.68 (*)
	EQ5D				0.07495 (n.s.)	0.1702 (*)	0.1733 (*)	0.5187 (*)
	BDI				− 0.8947 (n.s.)	− 2.053 (n.s.)	− 3.684 (*)	− 5579 (**)
40 Hz	VAS	3.632 (**)	− 1.000 (n.s.)	− 1.211 (*)		− 0.9474 (n.s.)	− 0.8421 (n.s.)	− 3.632 (**)
	MGPQ			− 2.263 (n.s.)		− 2.789 (n.s.)	− 3.211 (n.s.)	− 13.42 (*)
	EQ5D			0.07495 (n.s.)		0.09526 (n.s.)	0.09837 (n.s.)	0.4438 (*)
	BDI			− 0.8947 (n.s.)		− 1.158 (n.s.)	− 2.789 (n.s.)	− 4.684 (*)
60 Hz	VAS	2.684 (**)	− 1.947 (*)	− 2.158 (**)	− 0.9474 (n.s.)		0.1053 (n.s.)	− 2.684 (**)
	MGPQ			− 5.053 (n.s.)	− 2.789 (n.s.)		− 0.4211 (n.s.)	− 10.63 (*)
	EQ5D			0.1702 (*)	0.09526 (n.s.)		0.0031 (n.s.)	0.3485 (*)
	BDI			− 2.053 (n.s.)	− 1.158 (n.s.)		− 1.632 (n.s.)	− 3.526 (n.s.)
80 Hz	VAS	2.789 (**)	− 1.842 (*)	− 2.053 (**)	− 0.8421 (n.s.)	0.1053 (n.s.)		− 2.789 (**)
	MGPQ			− 5.474 (*)	− 3.211 (n.s.)	-0.4211 (n.s.)		− 10.21 (*)
	EQ5D			0.1733 (*)	0.09837 (n.s.)	0.0031 (n.s.)		0.3454 (*)
	BDI			− 3.684 (*)	− 2.789 (n.s.)	− 1.632 (n.s.)		− 1.895 (n.s.)
sham	VAS	0.000 (n.s.)	− 4.632 (**)	− 4.842 (**)	− 3.632 (**)	− 2.684 (**)	− 2.789 (**)	
	MGPQ			− 15.68 (*)	− 13.42 (*)	− 10.63 (*)	− 10.21 (*)	
	EQ5D			0.5187 (*)	0.4438 (*)	0.3485 (*)	0.3454 (*)	
	BDI			− 5579 (**)	− 4.684 (*)	− 3.526 (n.s.)	− 1.895 (n.s.)	

Although only assessed in a very basic fashion (increase in medication yes/no), the lowest number of patients reported an increased need for analgesic medication during 20 Hz stimulation (9 subjects), and 13 patients referred increased analgesics intake during 40 Hz stimulation and 16 subjects under 60 Hz and 80 Hz, whereas all 19 patients reported an increase during sham stimulation.

When stratified by PainDetect, a higher overall VAS and a higher mean difference in the VAS between stimulation frequencies were observed in the patients with a score > 12 without reaching statistical significance. The overall observation regarding better pain control with lower frequencies was still observed.

## Discussion

Dorsal root ganglion stimulation is an effective form of treatment for chronic, especially neuropathic, pain conditions. The choice of stimulation frequency shows a clear influence on pain reduction and the associated quality of life. Lower stimulation frequencies seem to be most effective in the examined pain etiologies, which is explained by the pathophysiology of pain processing.

A possible mechanism of action of DRG-S involves the activation of low-threshold mechanoreceptors, which are Aß-, Aδ-, and C-fiber afferents transmitting fine touch sensation. These fibers play an important role also inhibiting painful stimuli at the level of the dorsal horn [5]. Animal studies in vitro showed that high- and low-frequency DRG stimulations act over different inhibitory pathways in rats. Whereas low-frequency stimulation of 0.2–1.0 Hz promoted a pain relief that was suspended with naloxone, the effect of high-frequency stimulation of 100 Hz was reversed with GABA and glycine antagonists in transverse slices of rat spinal cords [6, 7, 13]. The different roles of high- and low-frequency DRG stimulations have not been investigated in humans so far.

The reason why low- and high-frequency stimulations may work differently is probably the phase locking of low-threshold mechanoreceptors. This occurs when neurons fire at the same frequency as the stimulation and it is only possible at certain stimulation frequencies depending on neurophysiological properties of each fiber. As shown by Arcourt et al. in a study with optogenetically modified rats, low-threshold mechanoreceptors in these animals were subject to phase locking for frequencies up to 20 Hz, after which neurons start asynchronous firing [1]. Assuming similar properties in the human population, for which such physiological studies lack, phase locking could be an explanation for the findings of the present study—the first of its type, to the best of our knowledge, with most patients reporting higher pain intensities under higher stimulation frequencies.

The frequency effect was less evident in patients with a PainDetect score under 12, which indicates a less pronounced neuropathic component in the overall pain. Dichotomizing the group by the PainDetect score did not result in a statistically significant difference but in a trend. This study might simply be underpowered to clearly reveal this difference. These subjects with an important nociceptive pain, which did also benefit from DRG-S in this trial like patients with classic neuropathic pain, seem not to rely exclusively on the endogenous intraspinal opioid inhibitory pathway for pain relief. This interesting finding is yet to be confirmed with further studies and could help extending neuromodulation for the much larger population with nociceptive pain.

In our study, we used a 2-day washout period. In most patients, DRG-S elicits fast to immediate response regarding pain control, but some effects of DRG-S go beyond pain control, e.g., autonomic symptoms in CRPS. These commonly take longer to become effective and are therefore likely underestimated in this study. For studies investigating only pain control, the washout period could even be shortened. In studies investigating autonomic effects, the stimulation interval and washout periods should be extended. This is especially important in studies looking into the efficacy of neuromodulation to modulate the function of immune system, e.g., to treat CRPS, osteoarthritis, and similar disorders [4].

This trial is the first to investigate the influence of stimulation frequencies in DRG-S in a double-blind, randomized, prospective setting. We tested frequencies down to 20 Hz—a mid-frequency stimulation. We recognize the potential of even lower stimulation frequencies down to 4 Hz, as shown by Chapman in his important case series [2]. We are currently further investigating the influence of stimulation frequency in DRG-S with the aim to predict optimal stimulation frequencies based in the underlying condition and the proportion of neuropathic and nociceptive pain. The relevance of such studies goes far beyond the expected elongation of battery lifetime; the focus is the targeted approach of different nerve fibers with unique neurophysiological properties. Additionally, stimulation with lower intensities and less energy-transfer is thought to induce less habituation preventing loss of effect over time [9].

### Limitations

The study results are limited by the fact that all the subjects were using 20 Hz of stimulation frequency for a long time prior to the beginning of the study.

## Conclusions

The choice of the stimulation frequency shows a clear influence on the pain reduction and the associated well-being and quality of life of the patient. Lower stimulation frequencies seem to be most effective for neuropathic pain. As soon as larger similar studies are available, conclusions will be drawn regarding the functioning of the DRG in different pain etiologies and the pathophysiology of pain processing.

## Abbreviations

DRG-S: Dorsal root ganglion stimulation
VAS: Visual analog scale
MPQ: McGill Pain Questionnaire
EQ-5D: EuroQol-5D
BDI: Beck Depression Inventory
CRPS: Complex regional pain syndrome
DRG: Dorsal root ganglion
DRKS: German Register of Clinical Studies
IPG: Implantable pulse generator
SD: Standard deviation
